# The E3 Ubiquitin Ligase Gene *Sl1* Is Critical for Cadmium Tolerance in *Solanum lycopersicum* L.

**DOI:** 10.3390/antiox11030456

**Published:** 2022-02-25

**Authors:** Chen-Xu Liu, Ting Yang, Hui Zhou, Golam Jalal Ahammed, Zhen-Yu Qi, Jie Zhou

**Affiliations:** 1 Zhejiang Provincial Key Laboratory of Horticultural Plant Integrative Biology, Department of Horticulture, Zhejiang University, Yuhangtang Road 866, Hangzhou 310058, China; chenxuliu@zju.edu.cn (C.-X.L.); 22116163@zju.edu.cn (T.Y.); 3180100610@zju.edu.cn (H.Z.); 2 College of Horticulture and Plant Protection, Henan University of Science and Technology, Luoyang 471023, China; 3 Agricultural Experiment Station, Zhejiang University, Hangzhou 310058, China; qizhenyu@zju.edu.cn; 4 Key Laboratory of Horticultural Plants Growth, Development and Quality Improvement, Agricultural Ministry of China, Yuhangtang Road 866, Hangzhou 310058, China; 5 Shandong (Linyi) Institute of Modern Agriculture, Zhejiang University, Linyi 276000, China

**Keywords:** antioxidant enzymes, heavy metal stress, ubiquitination, protein degradation, tomato

## Abstract

Heavy metal cadmium (Cd) at high concentrations severely disturbs plant growth and development. The E3 ubiquitin ligase involved in protein degradation is critical for plant tolerance to abiotic stress, but the role of E3 ubiquitin ligases in Cd tolerance is largely unknown in tomato. Here, we characterized an E3 ubiquitin ligase gene *Sl1*, which was highly expressed in roots under Cd stress in our previous study. The subcellular localization of Sl1 revealed that it was located in plasma membranes. In vitro ubiquitination assays confirmed that Sl1 had E3 ubiquitin ligase activity. Knockout of the *Sl1* gene by CRISPR/Cas9 genome editing technology reduced while its overexpression increased Cd tolerance as reflected by the changes in the actual quantum efficiency of PSII photochemistry (Φ_PSII_) and hydrogen peroxide (H_2_O_2_) accumulation. Cd-induced increased activities of antioxidant enzymes including superoxide dismutase (SOD), catalase (CAT), ascorbate peroxidase (APX), and glutathione reductase (GR) were compromised in *sl1* mutants but were enhanced in *Sl1* overexpressing lines. Furthermore, the content of Cd in both shoots and roots increased in *sl1* mutants while reduced in *Sl1* overexpressing plants. Gene expression assays revealed that Sl1 regulated the transcript levels of heavy metal transport-related genes to inhibit Cd accumulation. These findings demonstrate that Sl1 plays a critical role in regulating Cd tolerance by relieving oxidative stress and resisting heavy metal transportation in tomato. The study provides a new understanding of the mechanism of plant tolerance to heavy metal stress.

## 1. Introduction

The rapid development of industrialization and urbanization has resulted in severe environmental pollution [1]. Sewage or waste produced by industries and garbage generated by anthropogenic activities lead to the release of heavy metals into the environment, causing contamination of agricultural soil and water [2,3]. Thus, heavy metal pollution affects both human health as well as plant health, particularly plant growth and development [4].

Cadmium (Cd), a toxic heavy metal, severely inhibits plant growth and crop production when it occurs in high concentrations in soils or growth media [5]. The absorption and translocation of Cd in plants include distinct phases, such as the absorption of Cd in roots, the transportation of Cd into xylem and phloem, and the transportation of Cd into aboveground tissues [6]. The absorption and transportation of Cd in plants mainly depend on transport proteins such as heavy-metal-associated P-type ATPase family protein (HMA), ATP-binding cassette transporters (ABC), natural resistance-associated macrophage protein (NRAMP), metal-tolerance protein (MTP), calcium exchanger protein (CAX), and zinc/iron-regulated transporter-like protein (ZRT/IRT) [6,7,8,9,10,11,12].

Owing to the interaction with the sulfhydryl group of proteins, Cd can affect the activity of multiple enzymes and the functions of proteins [13]. Hence, the accumulation of Cd in plant tissues disorders various growth, biochemical, and physiological processes, such as photosynthesis, antioxidant enzyme activity, cell structure, and plant morphology [13,14]. Specifically, Cd stress causes damage to chloroplast ultrastructure, inhibits pigment synthesis, and affects several photosynthesis-related protein complexes [15]. Cd stress also impairs the balance between reactive oxygen species (ROS) production and ROS scavenging [16]. In particular, Cd stress disturbs the electron transfer chain in mitochondria and chloroplasts and activates NADPH oxidases, which cause excessive ROS accumulation and associated oxidative stress in plants [17,18]. Moreover, Cd stress induces protein denaturation, which disturbs the balance of protein quality in plant cells [19].

Plants have also evolved very diverse and complex defensive mechanisms for resisting heavy metal stress [20]. The ascorbate–glutathione (AsA–GSH) cycle is an indispensable pathway for eliminating oxidative stress [21]. Moreover, antioxidant enzymes including superoxide dismutase (SOD), catalase (CAT), ascorbate peroxidase (APX), peroxidase (POD), and glutathione reductase (GR) are vital for scavenging ROS [22]. Antioxidant GSH not only functions on relieving oxidative stress, but also participates in the synthesis of phytochelatins (PCs) in plants, which can bind with heavy metal ions [20,23,24]. Moreover, the protein quality control system needs chaperone proteins to refold the denatured proteins or operates a protein degradation system to clear misfolded proteins under Cd stress [24,25].

Ubiquitin proteasome system (UPS) has been recognized as a critical process to control the abundance, quality, and function of protein in cells [26,27]. Ubiquitin is an indispensable component of UPS, which acts as identifying target protein for degradation [28]. Ubiquitin-dependent protein degradation requires reactions of multiple ubiquitin-related enzymes, including ubiquitin activating enzyme (E1), ubiquitin conjugating enzyme (E2), and ubiquitin ligase (E3) [29,30]. The ubiquitin molecule is activated by E1 and then transferred to E2. The E3 interacts with E2-ubiquitin and targets proteins for labeling substrate protein with ubiquitin. Finally, the 26S proteasome degrades the target proteins [31,32,33]. E3 ubiquitin ligases play critical roles in recognizing substrate proteins [29]. Among the ubiquitin-related enzymes, E3 ubiquitin ligases have the largest family in plants [19]. The E3 ligase has been divided into three types, as follows: really interesting new gene (RING)-type, homology to E6-associated carboxyl-terminus (HECT)-type, and U-box-type [31].

RING-type E3 ubiquitin ligases have a cysteine-rich domain that can bind with Zn ions [31]. Previous studies have revealed that RING-type E3 ubiquitin ligases are involved in mediating plant tolerance of heavy metal stress [19,25,34]. A RING-type E3 ubiquitin ligase HIR1 confers tolerance of arsenic (As) and Cd stress in rice [34]. Overexpression of *HIR1* increases root length in rice under As and Cd treatment. The E3 ubiquitin ligase protein HIR1 also interacts with tonoplast intrinsic protein TIP4;1 and regulates its abundance in rice, thereby alleviating heavy metal stress [34]. Tomato E3 ubiquitin ligase RING1 increases Cd tolerance by minimizing ROS levels due to enhanced antioxidant enzyme activities [19,25]. However, the roles of many tomato E3 ubiquitin ligases are largely unknown, particularly in Cd stress.

In a previous study, we found that the expression of the RING-type E3 ubiquitin ligase *Sl1* significantly increased in tomato roots when challenged with aluminum (Al) or Cd stress [25]. However, the precise role of *Sl1* remains elusive. We hypothesized that *Sl1* might play a crucial role in Cd tolerance in tomato plants. Here, we characterized the function of *Sl1* by generating *sl1* mutants and *Sl1* overexpressing lines in tomato. Our study unveils a novel role of *Sl1* in plant tolerance to Cd stress.

## 2. Materials and Methods

### 2.1. Plant Materials and Treatments

In this study, tomato (*Solanum lycopersicum* L., Tomato Genetics Resource Center, Davis, CA, USA, https://tgrc.ucdavis.edu (accessed on 4 March 2018)) cultivar “Ailsa Craig” was used to generate *sl1* mutants and *Sl1* overexpressing transgenic lines.

Tomato seedlings were raised in vermiculite and transferred to a hydroponic jar (40 cm × 25 cm × 15 cm) containing Hoagland nutrient solution, when two real leaves of seedlings unfolded. The growth conditions were temperature of 23/20 °C (day/night), 14 h photoperiod, 60% humidity, and photosynthetic photo flux density (PPFD) of 600 μmol m^−2^ s^−1^. For Cd treatment, seedlings at the five-leaf stage were treated with 100 μM CdCl_2_ and the hydroponic nutrient solution was changed every 5 days (d).

### 2.2. Generation of Transgenic Plants

The *sl1* mutant was generated by CRISPR/Cas9 technique. For CRISPR vector construction, the target sequence used as sgRNA on the only exon of *Sl1* was searched by the CRISPR-P program (http://cbi.hzau.edu.cn/cgi-bin/CRISPR (accessed on 10 May 2019)). For deletion of a large fragment, two targets were selected (sgRNA-*Sl1*-254: TCATTAAAGGGTCTTCAACA and sgRNA-*Sl1*-564: GTTGAACTTGGAGCAATGAT), primers were then generated by adding adapter sequence to two ends of target sequences (Figure S1A). The sense and antisense sgRNA primers were annealed and inserted into the *Bbs*I site of the AtU6-sgRNA-AtUBQ-Cas9 vector, then positive clones were confirmed by sequencing. The two clones were named sgRNA-*Sl1*-254 and sgRNA-*Sl1*-564. The fragment was amplified using the clone of sgRNA-*Sl1*-564 as a template, the fragment was then inserted into the backbone vector with Cas9 and sgRNA-254. pCAMBIA1301 was used as a binary expression vector, and the sgRNA-254-sgRNA-564-Cas9 was inserted into the *EcoR*I and *Hind*III site of pCAMBIA1301. Positive clones were transformed into *Agrobacterium tumefaciens* strain GV3101 for transgenic plant generation.

For the construction of *Sl1* overexpressing transgenic lines, the full-length coding sequences (CDS) of *Sl1* were amplified with primers (Forward primer 5′-TTACAATTACCATGGGGCGCGCCATGGATCTTGTTAGACTAAAGTATTTTGAA-3′, Reverse verse primer 5′-AACATCGTATGGGTAGGTACCTGACTCTAACTGAATAGGTAAAACTACATTTC-3′), then inserted the PCR products into the *Asc*I and *Kpn*I site of pFGC1008-HA vector. The positive vector was confirmed by sequencing and then transformed into *A. tumefaciens* strain GV3101.

The detailed method of generation of transgenic lines was described previously [35]. Two independent homozygous lines of the F2 generation of *Sl1* overexpressing plants were used in further experiments. Two homozygous lines of *sl1* mutants without CRISPR/Cas9 DNA were selected for further research. The special primers for mutant detection were designed as follows: Forward primer 5′-GCAGAGAGACAACATTCACCA- 3′, Reverse primer 5′-AAAGTTGTCGATCCGTCGCT-3′.

### 2.3. E3 Ubiquitin Ligase Activity Assay

The full length CDS of *Sl1* were amplified with the primers (Forward primer 5′-GAGGGAAGGATTTCAGAATTCATGGATCTTGTTAGACTAAAGTATTTTGAA-3′, Reverse primers 5′-CAGGTCGACTCTAGAGGATCCTGACTCTAACTGAATAGGTAAAACTACATTTC-3′). The PCR products were digested with restriction endonuclease *EcoR*I and *BamH*I and were then inserted into the pMAL-2c vector (New England Biolabs, Ipswich, MA, USA). The maltose-binding protein-empty vector (MBP-EV) and MBP-fused Sl1 protein were expressed in *Escherichia coli* strain BL21 (DE3) and purified with instructions of the manufacturer (New England Biolabs, Ipswich, MA, USA). The in vitro ubiquitination assay of Sl1 was performed by instructions described previously [36]. The reaction system was prepared as described previously [19].

After the reaction, a Western blot was used to detect whether the Sl1 protein has E3 ubiquitin ligase activity. Anti-His (A5C12; HUABIO, Hangzhou, China) and anti-MBP (MBP61R; Thermo Fisher Scientific, Waltham, MA, USA) antibodies were used in Western blot assay.

### 2.4. Vector Construction and Subcellular Localization of Sl1

The full length CDS of *Sl1* were amplified with primers (Forward primer 5′-CTCTCGAGCTTTCGCGAGCTCATGGATCTTGTTAGACTAAAGTATTTTGAA-3′, Reverse primer 5′-GCCCTTGCTCACCATGGATCCTGACTCTAACTGAATAGGTAAAACTACATTTC-3′), and the PCR products were inserted into *Sac*I and *BamH*I site of pCAMBIA2300 with a GFP tag at the C terminus. The primers were designed by homologous recombination methods as described previously. After confirming positive clones by sequencing, the vector (35S-*Sl1*-GFP) and empty vector were transformed into *A. tumefaciens* strain GV3101. Transgenic *Nicotiana benthamiana* that expressed with nucleus-located signaling (RFP-H2B) was used for transient expression. The transient expression method was described previously [37]. For the fluorescence image acquisition, a Nikon A1 confocal microscope (Nikon, Tokyo, Japan) was used at 48 h post infiltration in tobacco leaves, and the excitation/emission wavelengths of GFP were 488 nm/480–520 nm, and the excitation/emission wavelengths of RFP were 561 nm/610–630 nm.

### 2.5. Measurement of Actual Quantum Efficiency of PSII Photochemistry

After 15 d treatment of Cd, plants were dark-adapted for 30 min before measurement. The actual quantum efficiency of PSII photochemistry (Φ_PSII_) was determined by the Imaging PAM (IMAG-MAXI, Heinz Walz, Germany) in the fifth fully expanded leaves as described previously [38].

### 2.6. Measurements of Hydrogen Peroxide and Antioxidants Enzyme Activity

Three-tenths gram of tomato root samples were collected and ground in liquid nitrogen. The samples were combined with 3 mL precooled 1 M HClO_4_ in a 10 mL centrifuge tube and mixed thoroughly. The pH of the sample was adjusted to 6–7 using 4 M KOH, 0.05 g activated carbon was then added to absorb pigments. The samples were centrifuged at 12,000× *rpm* for 10 min at 4 °C and supernatants were collected in 5 mL tubes for reaction. The reaction buffer was as follows: 1 mL supernatant or H_2_O_2_, 996 μL 1 mM ABTS (dissolved in 100 mM potassium acetate, pH 4.4), and 4 μL POD (P8375, Merck KGaA, Darmstadt, Germany). The absorbance of reaction buffer was detected at a wavelength of 412 nm by a SHIMADZU UV-2410PC spectrophotometer (Shimadzu Company, Kyoto, Japan) and the content of H_2_O_2_ was calculated by a standard curve [39].

Three-tenths gram fresh tomato roots were ground in liquid nitrogen and dissolved in a precooled buffer that included 50 mM phosphate buffered saline (pH 7.8), 0.2 mM EDTA, 2 mM ascorbic acid, and 2% (*w*/*v*) poly-vinylpolypyrrolidone. The samples were mixed thoroughly using vortex and then centrifuged at 12,000× *g* for 20 min at 4 °C. The supernatant was collected in new centrifuge tubes for detecting enzyme activity. The method of measuring enzyme activities of SOD, CAT, APX, and GR was described previously [40].

### 2.7. Measurement of Cd Content and Cd Localization

Tomato leaves and roots were washed and collected in liquid nitrogen after 10 d Cd treatment. Samples were put in the oven for 30 min at 115 °C then transferred to 60 °C for total dryness. The dried sample was ground and mixed with an acid solution that contained HClO_4_ and HNO_3_ (1:3, *v*:*v*). The samples were digested at 180 °C and the solution was then evaporated to 1-2 mL for dilution. The remained solution was diluted with deionized water to a final volume of 50 mL and inductively coupled plasma mass spectrometry (ICPMS-2030, Shimadzu Company, Kyoto, Japan) was used for determining Cd content [25].

For Cd localization, the Leadmium^TM^ Green AM probe (Invitrogen, Carlsbad, CA, USA) was used for Cd staining according to the manufacturer’s instructions. The root tips were stained by immersing in dye solution for 3 h in dark at room temperature; the sample was then washed three times with buffer (0.85% NaCl). A Nikon A1 confocal microscope (Nikon, Tokyo, Japan) was used to detect the Cd localization, and the excitation/emission wavelengths of GFP were 488 nm/510–530 nm [41]. The mean fluorescence intensity values of Cd stained root tips were detected by ImageJ 1.53 analysis software (National Institute of Health, Bellevue, WA, USA) and relative fluorescence intensity normalized to the intensity of the wild-type group under Cd stress.

### 2.8. Total RNA Isolation and qRT-PCR Analysis

One-tenth gram tomato samples were collected in a 2 mL centrifuge tube and frozen in liquid nitrogen. An RNAprep pure plant kit (Tiangen Biotech, Beijing, China) was used for total RNA extraction according to the manufacturer’s instructions. Five-tenths milligram total RNA was used to reverse transcribe to cDNA template by HiScript II Q RT SuperMix for qPCR Kit (Vazyme, Nanjing, China). qRT-PCR was conducted with ChamQ Universal SYBR qPCR Master Mix (Vazyme, Nanjing, China) on a Light Cycle 480 II Real-Time PCR detection system (Roche, Basel, Switzerland). The total 20 μL reaction system was as follows: 10 μL SYBR qPCR Master Mix, 1 μL cDNA template, 10 μM forward and reverse primer, and deionized water. The PCR program was performed with 30 s at 95 °C, followed with 35–40 cycles of 10 s at 95 °C, 30 s at 58 °C, and 1 min at 72 °C. *ACTIN* was used for calculating the relative expression level of the target gene. The primers are listed in Table S1.

### 2.9. Immunoblotting Assays

The protein extraction and Western blot procedures were described previously [42,43]. The antibodies used in this assay included anti-HA (26183, Thermo Fisher Scientific, Waltham, MA, USA) and rabbit antimouse (ab6728, abcam, Cambridge, UK). The signals were visualized with FDbio-Femto ECL (FD8030, Fdbio science, Hangzhou, China).

### 2.10. Statistical Analysis

All experiments were conducted independently three times and three replications of each experiment were performed. All data were analyzed by SPSS 16.0 statistical software package and Tukey’s test (*p* < 0.05) was used for significance analysis.

## 3. Results

### 3.1. Structure and Expression Analysis of Sl1

**Figure 1:**
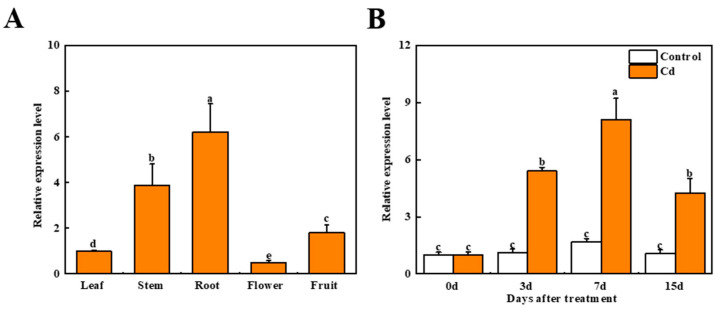
The relative expression level of *Sl1* in tomato plants. (**A**) The relative expression level of *Sl1* in different tissues of tomato. (**B**) Time course of the relative expression level of *Sl1* in tomato root with and without Cd stress. The data presented here are the average of three biological replicates (±SD). Different letters indicate a significant difference (*p* < 0.05, Tukey’s test).

The *Sl1* (Solyc09g089890) gene that was previously reported as one of the highly expressed genes in tomato roots under Cd stress was selected as the target gene in the current study [25]. We examined the time course of the relative expression level of *Sl1* in roots exposed to Cd stress. After 7 d treatment, the expression of *Sl1* reached the peak, which was 4.9-fold of that in control (Figure 1). Moreover, we examined the relative expression of the *Sl1* gene in different tissues including leaf, stem, root, flower, and fruit. The results showed that the relative expression level of *Sl1* in roots was 6-fold of that in leaves and 1.6-fold of that in stems (Figure 1).

The *Sl1* gene locates on chromosome 9 with only one exon on coding sequences (CDS) that encodes 349 amino acids. We used InterPro (https://www.ebi.ac.uk/interpro/ (accessed on 20 October 2021)) and Expasy (https://web.expasy.org/compute_pi/ (accessed on 22 October 2021)) to analyze the structure of the Sl1 protein. The results show that the isoelectric point and molecular weight of Sl1 protein are 8.56 and 40.14 kDa, respectively, and Sl1 contains a RING domain of 127 to 170 amino acids (Figure S2A,B).

### 3.2. E3 Ubiquitin Ligase Activity and Subcellular Localization of Sl1

**Figure 2:**
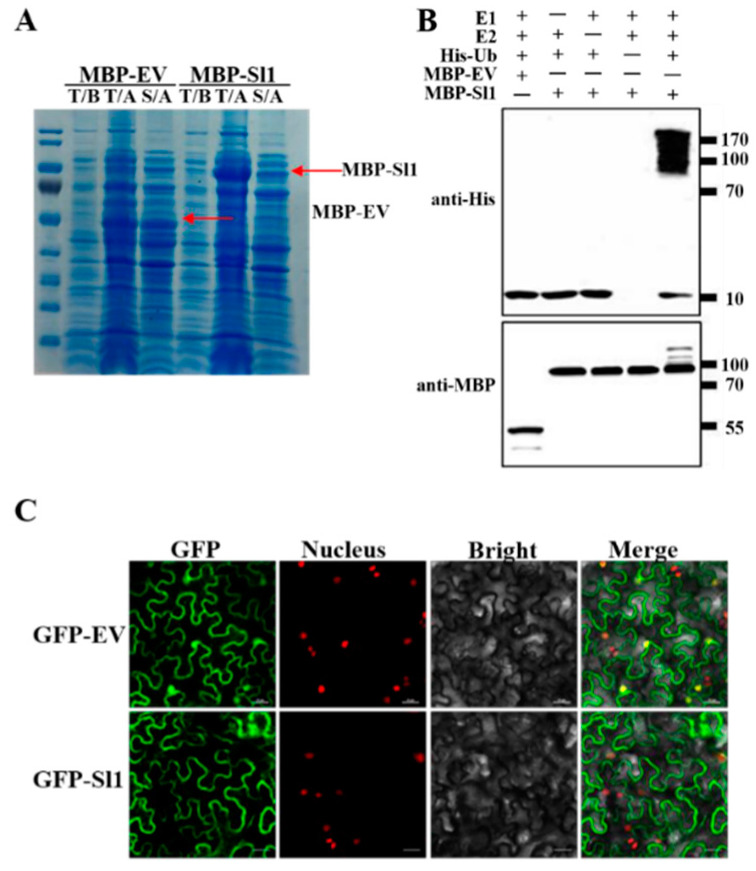
E3 ligase activity and subcellular localization of Sl1. (**A**) In vitro expression of MBP-Sl1 and MBP-EV. T: total protein; S: soluble protein; B: before expression; A: after expression. (**B**) In vitro E3 ligase activity of Sl1 protein. The reaction system included E1, E2, MBP-Sl1, and ubiquitin-His, the replacement of MBP-Sl1 with MBP-EV and the absence of E1, E2, and His-Ub as control. The Western blot was detected with anti-MBP and anti-His. (**C**) Subcellular localization of GFP-Sl1 and GFP-EV. The GFP-Sl1 was transiently expressed in *Nicotiana benthamiana* (tobacco with nucleus-located mCherry). Images were pictured by confocal microscope after 48 h infiltration. Bar = 25 μm.

To verify the E3 ubiquitin ligase activity of Sl1 protein, we successfully purified the maltose-binding protein-Sl1 (MBP-Sl1) fusion protein and maltose-binding protein-empty vector (MBP-EV) fusion protein following the manufacturer instructions (Figure 2A). We performed the in vitro ubiquitination assay and showed that Sl1 protein had self-ubiquitination ability when present with E1, E2, and ubiquitin. However, lacking any of E1, E2, or ubiquitin in the reaction system undermined the self-ubiquitination ability of Sl1. Meanwhile, MBP-EV also did not show self-ubiquitination ability (Figure 2B).

To examine the subcellular localization of Sl1 protein, we transiently expressed GFP-Sl1 in transgenic tobacco (with nucleus signal). Images captured by a confocal microscope revealed that the Sl1 protein is located in plasma membranes rather than the nucleus (Figure 2C).

### 3.3. Sl1 Positively Regulates Cd Tolerance in Tomato

**Figure 3:**
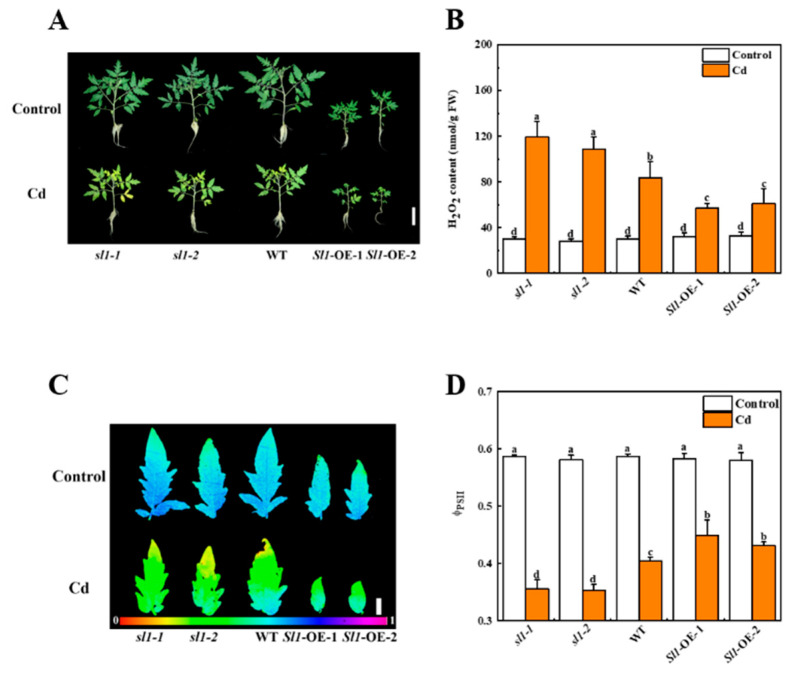
Sl1 positively regulates tomato Cd tolerance. (**A**) The phenotype of *Sl1* mutant lines (*sl1-1/2*), wild-type (WT), and *Sl1* overexpressing lines (*Sl1*-OE-1/2) under control and Cd stress after 15 d treatment. Bar = 10 cm. (**B**) The content of hydrogen peroxide (H_2_O_2_) in the roots of *Sl1* mutant lines (*sl1-1/2*), WT, and *Sl1* overexpressing lines (*Sl1*-OE-1/2) under control and Cd stress after 3 d treatment. (**C**,**D**) the image and level of actual quantum efficiency of PSII photochemistry (Φ_PSII_) of *sl1-1/2*, WT, and *Sl1*-OE-1/2 plants with and without Cd treatment for 15 d. Bar = 1 cm. The data presented here are the average of three biological replicates (±SD). Different letters indicate a significant difference (*p* < 0.05, Tukey’s test).

Since the expression of the *Sl1* gene was induced by Cd stress, we investigated the tolerance of *sl1* mutant lines, wild-type, and *Sl1* overexpressing lines to Cd stress. We generated the *sl1* mutant lines and *Sl1* overexpressing lines as described in the Methods and Materials section (Figure S1). Two *sl1* mutant lines were mutated at different sites that both induced early termination of translation. The *sl1-1* mutant line was found 311 bp deletion between sgRNA1 and sgRNA2 and the translation was terminated after 98 amino acids (Figure S1B). The *sl1-2* mutant line was deleted 1 bp after the protospacer adjacent motif (PAM) of sgRNA1 and the translation was terminated after 89 amino acids (Figure S1B). Two overexpressing lines of *Sl1* were checked with Western blot that both had bright bands near the predictive molecular weight (Figure S1C). The phenotypes of *sl1* mutants were similar to wild-type plants; however, *Sl1* overexpressing lines grew more slowly and showed smaller leaves compared with wild-type plants when they were grown under optimal (nonstress) environments (Figure 3A).

After 15 d of Cd treatment, *sl1* mutants showed sensitivity to Cd stress, while *Sl1*-OE showed enhanced tolerance to Cd stress (Figure 3). Compared to control conditions, Cd-stress-induced changes in leaf size, leaf color, level of Φ_PSII_, and content of hydrogen peroxide (H_2_O_2_) in *sl1* mutants and wild-type plants. In particular, the leaves of *sl1* were more etiolated than those of wild-type, and the leaf sizes of mutants were smaller than that of wild-type. However, the leaf size and color in *Sl1* overexpressing plants were just slightly affected by Cd stress (Figure 3). Since H_2_O_2_ is a major ROS generated under stress conditions, we then detected the accumulation of H_2_O_2_ in roots under Cd stress. Importantly, the accumulation of H_2_O_2_ increased by 43.1% and 30.5% in *sl1-1* and *sl1-2* mutants, while it decreased by 31.7% and 26.5% in two lines of *Sl1* overexpressing plants compared with wild-type plants after Cd treatment, respectively (Figure 3B). Moreover, we detected the value of actual quantum efficiency of PSII photochemistry, Φ_PSII_, which reflects the state of photosystem II as a reliable marker of plant health status. As shown in Figure 3C,D, the Φ_PSII_ value of *sl1* mutants, wild-type, and *Sl1* overexpressing lines exhibited no significant difference under control conditions. However, the Φ_PSII_ levels of *sl1-1* and *sl1-2* mutants decreased by 12.1% and 12.7% respectively, compared with wild-type under Cd stress (Figure 3D). The Φ_PSII_ levels of two lines of *Sl1* overexpressing plants were significantly greater than that in wild-type under Cd stress (Figure 3C,D). These results indicate that Sl1 is critical for alleviating Cd-induced H_2_O_2_ accumulation and damage to the photosynthetic system.

### 3.4. Sl1 Promotes Antioxidant Enzyme Activity

**Figure 4:**
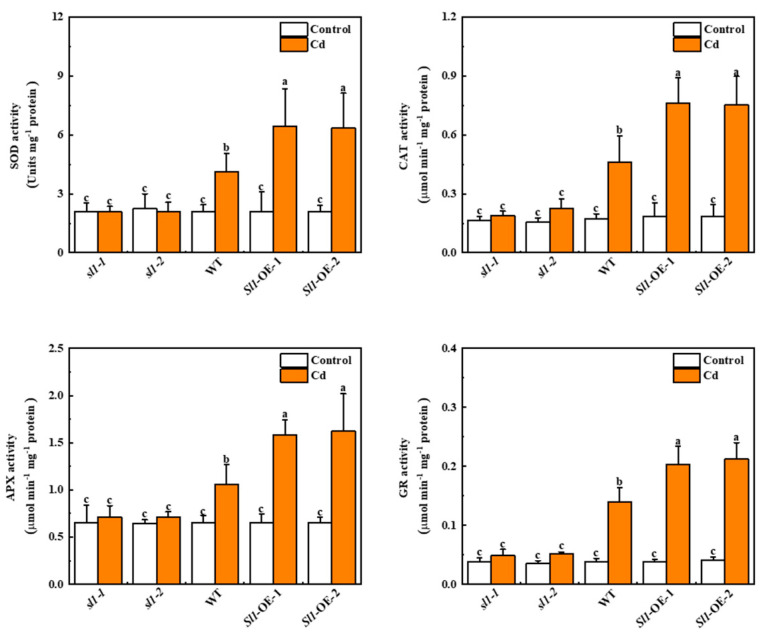
Sl1 promotes antioxidant enzyme activities in tomato plants under Cd stress. The activities of SOD, CAT, APX, and GR in the roots of *Sl1* mutant lines (*sl1-1/2*), wild-type (WT), and *Sl1* overexpressing lines (*Sl1*-OE-1/2) under Cd stress for 3 d. The data presented here are the average of three biological replicates (±SD). Different letters indicate a significant difference (*p* < 0.05, Tukey’s test).

To understand whether *Sl1* influenced antioxidant enzyme activities in tomato under Cd stress, we examined the enzyme activities of superoxide dismutase (SOD), catalase (CAT), ascorbate peroxidase (APX), and glutathione reductase (GR). The results showed that Cd stress increased antioxidant enzyme activities in wild-type and overexpressing lines. However, in *sl1* mutant lines, there were no significant differences in antioxidant enzyme activities between control and Cd treatment (Figure 4). The activities of SOD, CAT, APX, and GR in two lines of *Sl1* overexpressing plants were all induced compared with wild-type plants under Cd stress (Figure 4). These results suggest that *Sl1* promotes the activities of antioxidant enzymes under Cd stress.

### 3.5. Sl1 Reduces Cd Accumulation and Transportation

**Figure 5:**
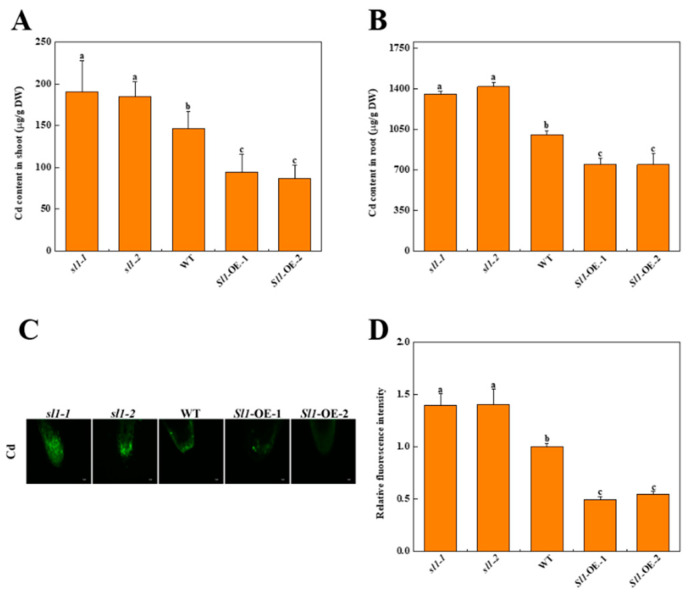
Sl1 decreases Cd content in tomato plants under Cd stress. The Cd content in the shoot (**A**) and root (**B**) of *Sl1* mutant lines (*sl1-1/2*), wild-type (WT), and *Sl1* overexpressing lines (*Sl1*-OE-1/2) under Cd stress for 10 d. (**C**) Cd accumulation in tomato root tips stained by the Cd-specific probe Leadmium^TM^ Green AM. Bar = 25 μm. (**D**) Relative fluorescence intensity of Cd staining over tomato root tips of *sl1* mutants, wild-type, and *Sl1* overexpressing lines after 10 d Cd treatment. The relative fluorescence intensity is normalized to the intensity of wild-type in (**C**). The data presented here are the average of three biological replicates (±SD). Different letters indicate a significant difference (*p* < 0.05, Tukey’s test).

To investigate whether *Sl1* is involved in Cd accumulation in tomato plants, we detected the content of Cd in shoots and roots under Cd stress. Results revealed that the Cd content in the roots was higher than that in the shoots (Figure 5A,B). Obviously, overexpression of *Sl1* decreased Cd content in both shoots and roots, while Cd content in *sl1* mutants significantly increased compared with that in wild-type (Figure 5A,B). The content of Cd in roots of two *Sl1* overexpressing lines both decreased by 25.6% compared with wild-type, while the Cd content increased by 34.7% and 41.6% in roots of *sl1-1* and *sl1-2* mutants compared with wild-type, respectively. Similarly, Cd accumulation was also higher in the shoots of *sl1* mutants than wild-type plants, while it was lower in the shoots of *Sl1* overexpressing lines.

To further investigate whether *Sl1* decreased Cd accumulation by altering Cd delivery, we used a Cd-specific probe to study the Cd distribution in the root tips. The Cd-specific probe stained signals were not detected in the root tips of all plants without Cd treatment (Figure S2E). However, Cd treatment induced the accumulation of Cd in the root tips as reflected by the increased fluorescence intensity. The relative fluorescence intensity of *sl1-1* and *sl1-2* mutants were 1.40-fold and 1.41-fold of that in wild-type plants, respectively, while the relative fluorescence intensity of *Sl1*-OE-1 and *Sl1*-OE-2 plants were only 49.2% and 54.7% of that in wild-type plants (Figure 5C,D).

**Figure 6:**
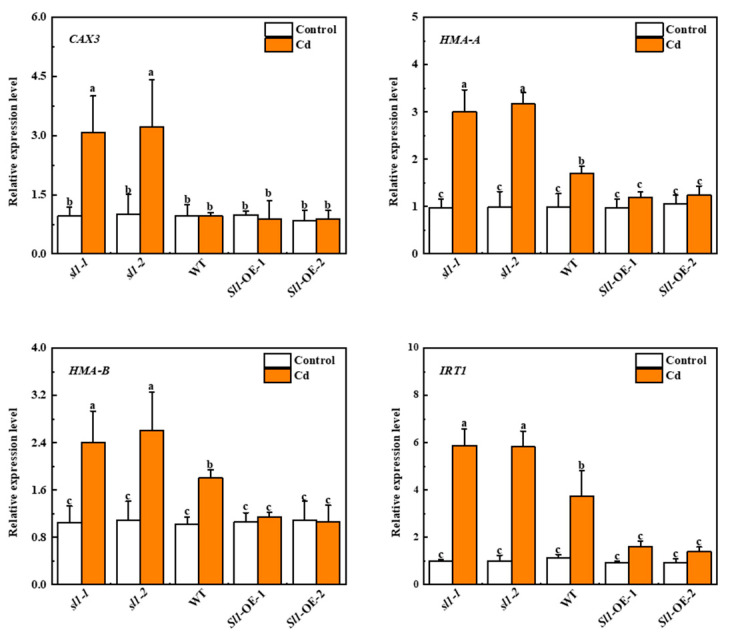
Sl1 negatively regulates the transcripts of genes related to heavy metal transportation. The relative expression of *CAX3*, *HMA-A*, *HMA-B,* and *IRT1* in the roots of *Sl1* mutant lines (*sl1-1/2*), wild-type (WT), and *Sl1* overexpressing lines (*Sl1*-OE-1/2) under Cd stress for 3 d. The data presented here are the average of three biological replicates (±SD). Different letters indicate a significant difference (*p* < 0.05, Tukey’s test).

To investigate how *Sl1* regulated Cd transportation, we examined the expression of heavy metal transportation-related genes (*CAX3*, *HMA-A*, *HMA-B*, and *IRT1*). There was no significant difference in the expression of these four genes between *sl1* mutants, wild-type, and *Sl1* overexpressing lines under control conditions. Although these four genes were highly expressed in *sl1* mutants under Cd stress, no significant difference was found between control and Cd treatment in two lines of *Sl1* overexpressing plants. We found that Cd stress dramatically increased the transcript level of *CAX3* in *sl1-1* and *sl1-2* mutants under Cd stress, which were 3.2-fold and 3.4-fold of that in wild-type, respectively (Figure 6). The heavy metal transport gene *HMA-A/B* was also upregulated by Cd stress in *sl1-1* and *sl1-2* plants by 76.5%/73.6% and 86.5%/79.9% compared with wild-type, respectively (Figure 6). Similarly, the expression of *IRT1*, which plays a prominent role in heavy metal transportation, was also increased by Cd stress in *sl1* mutants compared with wild-type plants (Figure 6). These results suggest *Sl1* potentially functions in resisting heavy metal transportation through repressing the transcription of heavy metal transportation-related genes.

## 4. Discussion

As a significant component of the food chain, plants play a crucial role in the transportation and accumulation of toxic elements such as Cd in humans [44]. Nonetheless, plants also suffer from the stress induced by heavy metals and they address the stress by multiple pathways, including eliminating ROS, resisting heavy metal transportation, and maintaining protein quality [19,20,24,25]. The E3 ubiquitin ligase-mediated protein degradation plays an important role in plant stress tolerance [25,34,36,45,46]. Here, we characterized a RING-type E3 ubiquitin ligase Sl1, which conferred Cd tolerance in tomato. Our study advances the understanding of the mechanism of UPS-mediated heavy metal tolerance in plants.

The RING-type E3 ligase is closely associated with plant tolerance to various stress [47]. For example, a C3H2C3-type E3 ubiquitin ligase AtAIRP1 positively regulates ABA-dependent drought tolerance by mediating AtAIRP1 degradation [48,49]. The roles of E3 ubiquitin ligases in the positive regulation of heavy metal stress tolerance have been reported recently in different plant species [19,25,34,46]. Overexpression of *HIR1* increases the tolerance of rice to As and Cd stress [34]. *HIR1* that encodes an E3 ubiquitin ligase interacts with TIP4;1 for resisting heavy metal absorption in rice [34]. Moreover, a U-box type E3 ubiquitin ligase, *SlUPS*, is highly expressed under Cd stress in tomato. Heterologous expression of *SlUPS* in yeast increases the concentration of yeast bacterial fluid exposed to Cd and overexpression of *SlUPS* in *Arabidopsis* enhances Cd tolerance [46]. The RING-type E3 ligase AtIDF1 degrades IRT1 to modulate iron homeostasis in *Arabidopsis* [50]. Heterologous expression of a soybean RING-type E3 ubiquitin ligase gene *GmARI1* in *Arabidopsis* enhances Al tolerance [51]. Furthermore, tomato E3 ubiquitin ligase SlRING1 positively mediates Cd tolerance by enhancing antioxidant enzyme activities and inhibiting Cd accumulation [19,25]. Similar to *SlRING1*, *Sl1*, which is highly expressed under Cd stress in tomato roots, plays a pivotal role in Cd tolerance (Figure 1 and Figure 3). We also found that Sl1 protein possessed the E3 ligase activity and overexpression of *Sl1* inhibited Cd accumulation in tomato. These results are consistent with previous studies [19,34], suggesting that RING-type E3 ubiquitin ligases play crucial roles in regulating metal ion transport.

Cadmium has a broad variety of negative impacts on plants, including oxidative stress, nutrient absorption disruption, and even plant mortality. Antioxidant enzymes such as SOD, POD, CAT, APX, and GR function in collaboration with nonenzymatic antioxidants such as AsA and GSH to prevent Cd-induced oxidative damage [52,53]. Furthermore, GSH directly participates in the synthesis of PCs [54,55]. PCs form complexes with Cd that can be compartmentalized into the vacuoles; thus, PCs and other thiols play an important role in determining the sensitivity or tolerance in contrasting genotypes of a plant species [56]. Moreover, studies on the semihalophytic plant *Mesembryanthemum crystallinum* L. and different Cd hyperaccumulators such as *Arabidopsis halleri*, *Thlaspi caerulescens*, *Solanum nigrum*, and *Sedum alfredii* species indicate that both antioxidative enzymes and nonenzymatic antioxidants play a vital role in Cd tolerance [57,58,59]. Previously, we found that an E3 ligase gene *SlRING1* positively regulates relative expression levels of *CAT*, *MDHAR*, *GSH1*, and *PCS*, while it decreases H_2_O_2_ content in tomato under Cd stress [19,25]. Consistent with those studies, *Sl1* overexpression increased the activities of SOD, CAT, APX, and GR, and decreased the content of H_2_O_2_ under Cd stress in *Sl1* overexpressing lines compared with those in wild-type and mutant lines (Figure 3 and Figure 4).

Metal absorption and transportation are critical for plant tolerance to heavy metal stress [44]. The rice E3 ubiquitin ligase OsHIR1 targets TIP4;1 that functions as a heavy metal absorption protein, and thus OsHIR1-induced degradation of TIP4;1 increases rice tolerance to As and Cd stress [34]. Moreover, another RING-type E3 ubiquitin ligase in rice, OsAIR3, regulates protein degradation of molybdate transporter (OsMOT1;3) in rice to increase plant tolerance to arsenate stress [60]. In agreement with these studies, we found that overexpression of *Sl1* significantly attenuated the relative expression level of several genes related to heavy metal transportation, such as *CAX3*, *HMA-A*, *HMA-B,* and *IRT1*, along with decreased Cd content in *Sl1* overexpressing lines compared with the wild-type and mutant lines (Figure 5 and Figure 6). *CAX* gene family is an important plant gene family involved in heavy metal transportation [11]. It is plausible that Sl1 functions as an E3 ubiquitin ligase for the degradation of proteins involved in heavy metal transporters or regulating the abundance of transcription factors upstream of those transporters. Interestingly, heavy-metal-induced stress also results in protein denaturation that aggravates the oxidative stress in plants [24]. Thus, further studies are essentially needed to investigate whether *Sl1* could degrade denatured proteins to relieve cell oxidative stress. Moreover, it will also be interesting to study whether *Sl1* can coordinate with autophagy to clear denatured proteins [61].

Plants do not have a Cd-selective transporter, therefore, Cd absorption happens through plasma membrane transporters that also take up other divalent cations [62]. Thus, it is indeed difficult to reduce plant Cd accumulation without compromising plant growth since the majority of well-known Cd transporters also transport various essential micronutrients such as Zn, Fe, Mn, and Cu. Despite the fact that CAXs are mostly Ca^2+^ specific transporters, AtCAX2 and AtCAX4 have been demonstrated to transport various other metals, such as Cd, Zn, and Mn in *Arabidopsis* [58]. Similarly, Fe-specific transporter OsIRT1 has been found to participate in Cd uptake in rice [63]. Thus, suppression of the metal transporter may lead to essential nutrient deficiency and compromised plant growth. In our study, overexpression of *Sl1* in tomato not only decreased Cd accumulation but also suppressed plant growth. Reduced Cd accumulation was associated with decreased expression of several metal transporters such as *CAX3*, *HMA-A*, *HMA-B*, and *IRT1*. Thus, it is possible that decreased plant growth in *Sl1* overexpressing lines could be a consequence of the suppression of the metal transporters under Cd stress. However, reduced growth of the *Sl1* overexpressing lines under control conditions could be attributed to some other reasons such as impaired hormone homeostasis since the expression levels of *CAX3*, *HMA-A*, *HMA-B*, and *IRT1* were not significantly different among *sl1* mutants, wild-type, and *Sl1* overexpressing lines under control conditions. Thus, it would be interesting to explore hormonal involvement in *Sl1*-regulated plant growth and stress tolerance in future studies.

## 5. Conclusions

In the present study, we characterized an E3 ubiquitin ligase Sl1 in tomato, which is located in plasma membranes and highly expressed in roots under Cd stress. For functional characterization of Sl1, we generated knockout lines and overexpressing lines of *Sl1* in tomato. The parameters of chlorophyll fluorescence and content of H_2_O_2_ demonstrated that *Sl1* overexpressing lines suffered less photosystem damage and oxidative stress compared with wild-type and mutants, suggesting that *Sl1* overexpressing lines are resistant to Cd stress, while mutant lines are sensitive to Cd stress. Moreover, Sl1 positively regulated antioxidant enzyme activities and negatively mediated gene expression associated with heavy metal transportation. Thus, the current study unveils a novel role of an E3 ubiquitin ligase Sl1 in tomato that may have potential implications in enhancing heavy metal tolerance in plants. However, identification of the substrate protein of Sl1 needs further study to precisely verify its association with heavy metal transportation.

## Data Availability

Data is contained within the article and Supplementary Materials.

## Supplementary Materials

The following supporting information can be downloaded at: https://www.mdpi.com/article/10.3390/antiox11030456/s1, Figure S1: Generation of *Sl1* mutant lines and overexpression lines; Figure S2: Structure analysis of Sl1 protein and histochemical staining of Cd accumulation of tomato root tips; Table S1: The primers used for qRT-PCR. Supplementary material related to this article can be found in the online version.
