# Supplementary material for: The E3 Ubiquitin Ligase Gene Sl1 Is Critical for Cadmium Tolerance in Solanum lycopersicum L
Source: Antioxidants (Basel). 2022 Feb 25;11(3):456. doi: 10.3390/antiox11030456 (PMC8944816; doi:10.3390/antiox11030456)
Supplement: Supplementary file 1 [file antioxidants-11-00456-s001.zip › Supplemental Figures.pptx]

## Slide 1
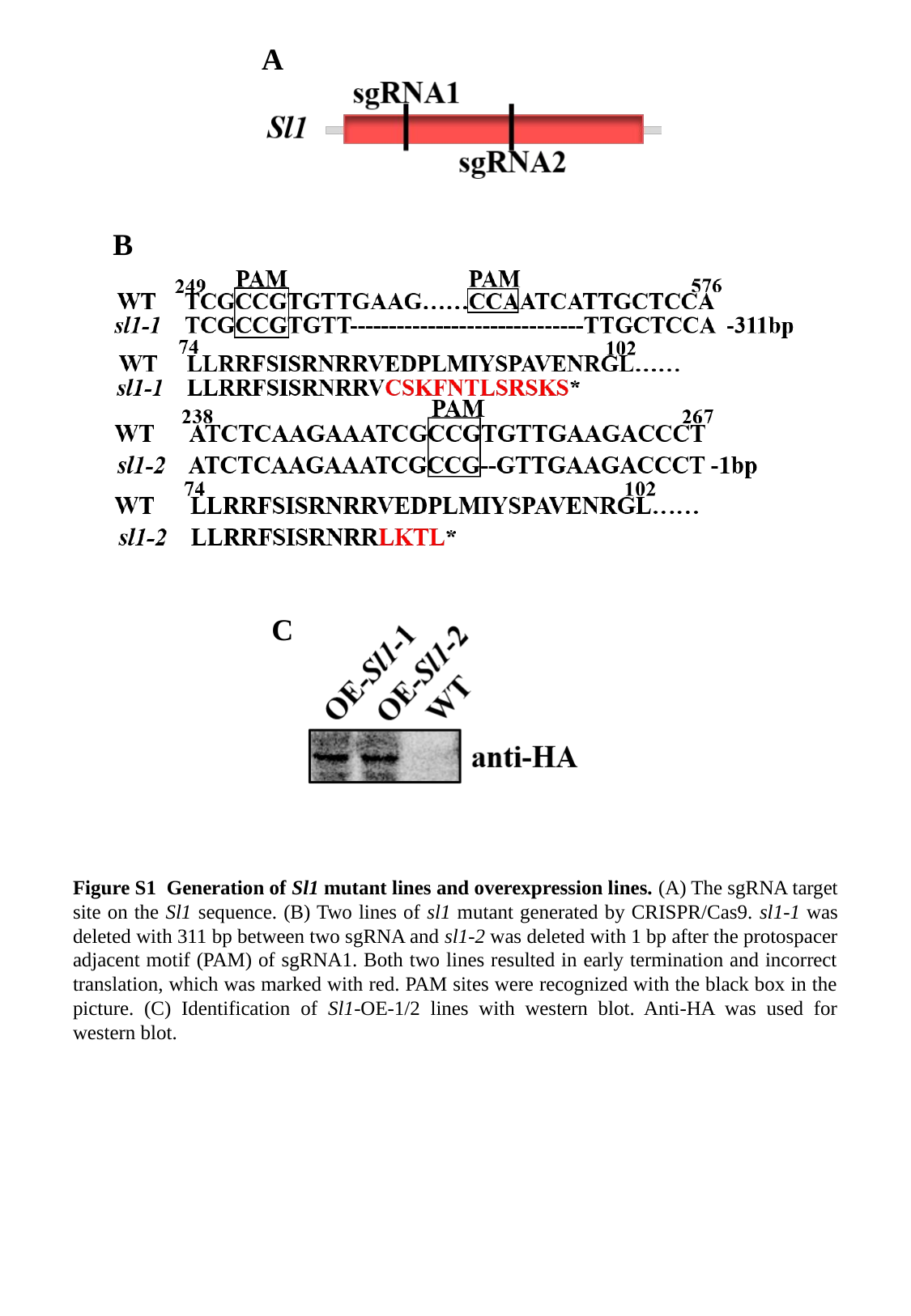

A
B
C
Figure S1 Generation of Sl1 mutant lines and overexpression lines. (A) The sgRNA target site on the Sl1 sequence. (B) Two lines of sl1 mutant generated by CRISPR/Cas9. sl1-1 was deleted with 311 bp between two sgRNA and sl1-2 was deleted with 1 bp after the protospacer adjacent motif (PAM) of sgRNA1. Both two lines resulted in early termination and incorrect translation, which was marked with red. PAM sites were recognized with the black box in the picture. (C) Identification of Sl1-OE-1/2 lines with western blot. Anti-HA was used for western blot.

## Slide 2
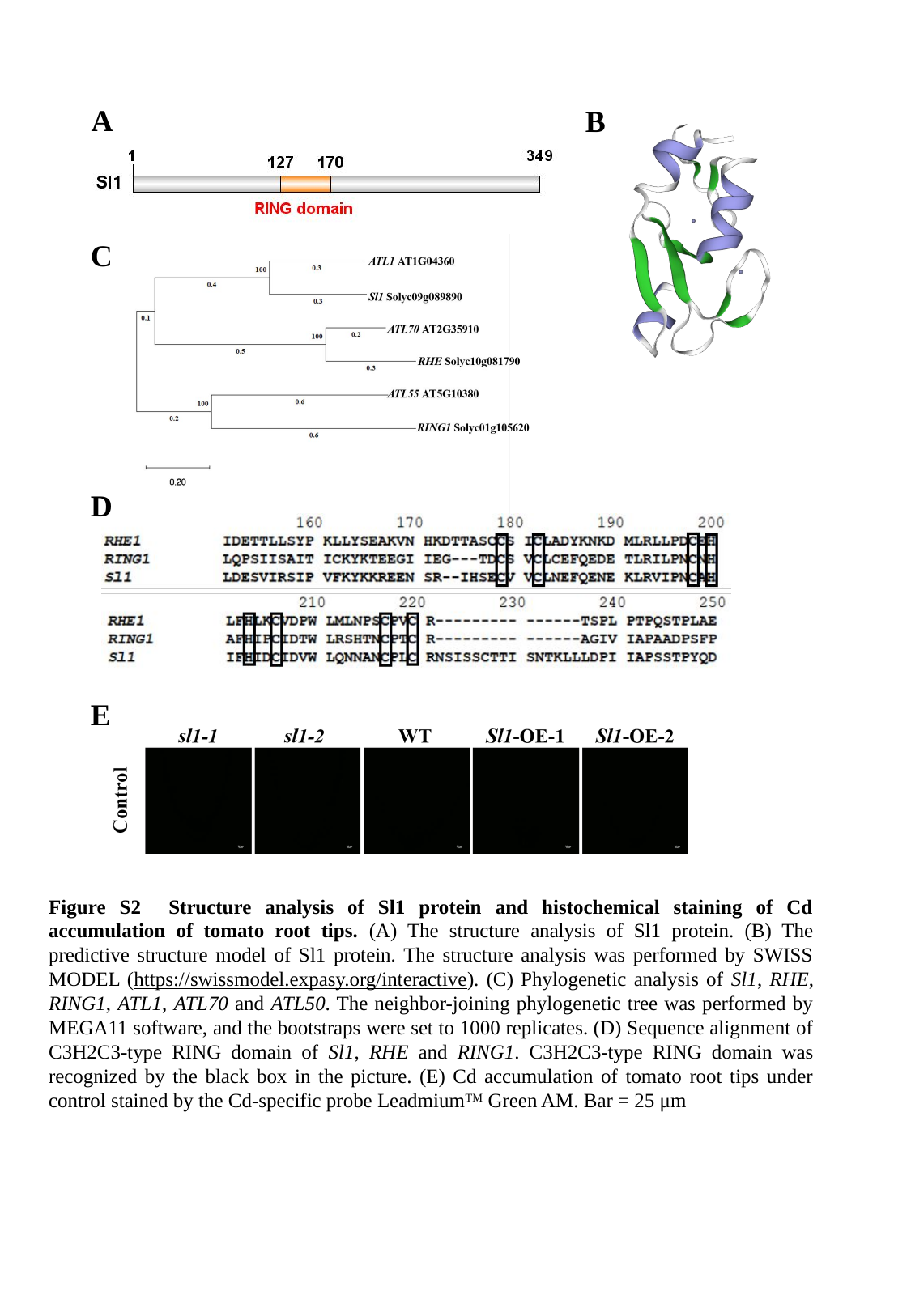

A
B
C
D
E
Figure S2 Structure analysis of Sl1 protein and histochemical staining of Cd accumulation of tomato root tips. (A) The structure analysis of Sl1 protein. (B) The predictive structure model of Sl1 protein. The structure analysis was performed by SWISS MODEL (https://swissmodel.expasy.org/interactive). (C) Phylogenetic analysis of Sl1, RHE, RING1, ATL1, ATL70 and ATL50. The neighbor-joining phylogenetic tree was performed by MEGA11 software, and the bootstraps were set to 1000 replicates. (D) Sequence alignment of C3H2C3-type RING domain of Sl1, RHE and RING1. C3H2C3-type RING domain was recognized by the black box in the picture. (E) Cd accumulation of tomato root tips under control stained by the Cd-specific probe LeadmiumTM Green AM. Bar = 25 μm
