# Supplementary material for: The E3 Ubiquitin Ligase Gene Sl1 Is Critical for Cadmium Tolerance in Solanum lycopersicum L
Source: Antioxidants (Basel). 2022 Feb 25;11(3):456. doi: 10.3390/antiox11030456 (PMC8944816; doi:10.3390/antiox11030456)
Supplement: Supplementary file 1 [file antioxidants-11-00456-s001.zip › Table S1.pdf]

Table S1 The primers used for qRT-PCR

Gene	Accession number	Forward primer (5'-3')	Reverse primer (5'-3')
CAX3	Solyc09g005260	GGAAATGCTGCTGAACATGC	AAGGGAACCACAAACATGGC
HMA-A	Solyc07g009130	GTGAAGGCAAAGAAGACATG	CTGCTCGCCAAACTTATCTA
HMA-B	Solyc02g068490	TTACTCGGTCTATTCGGTGT	GCTGTAATAATGAGGCTGTC
IRT1	Solyc02g069200	GCACTTGGGATAGCATTGTC	CACCCATAAAATCAGCAGCA
ACTIN	Solyc03g078400	TGTCCCTATTTACGAGGGTTATGC	CAGTTAAATCACGACCAGCAAGAT
Sl1	Solyc09g089890	CGACGGATCGACAACTTTAC	CCCTTCCATGTCCAAATGA
